# Supplementary material for: HIF1-alpha expressing cells induce a hypoxic-like response in neighbouring cancer cells
Source: BMC Cancer. 2018 Jun 20;18:674. doi: 10.1186/s12885-018-4577-1 (PMC6011406; doi:10.1186/s12885-018-4577-1)
Supplement: Supplementary file 1 — Figure S4. HIF activation by hypoxic and mimetic culture. A. Western blot was first labelled for HIF (Left), then washed and re-blotted for Tubulin (right). This unedited western shows MCF7 and MDA-MB-231 parental cells cultured in 21% and 1% oxygen for 48 hours before lysis. B. Western blot was cut at approximately 80kDa and the top was labelled for HIF1 whilst the bottom was labelled for Tubulin. This unedited western shows MCF7 and MDA-MB-231 parental cells exposed to varying concentrations of CoCl2. (PPTX 1037 kb). [file 12885_2018_4577_MOESM1_ESM.pptx]

## Slide 1
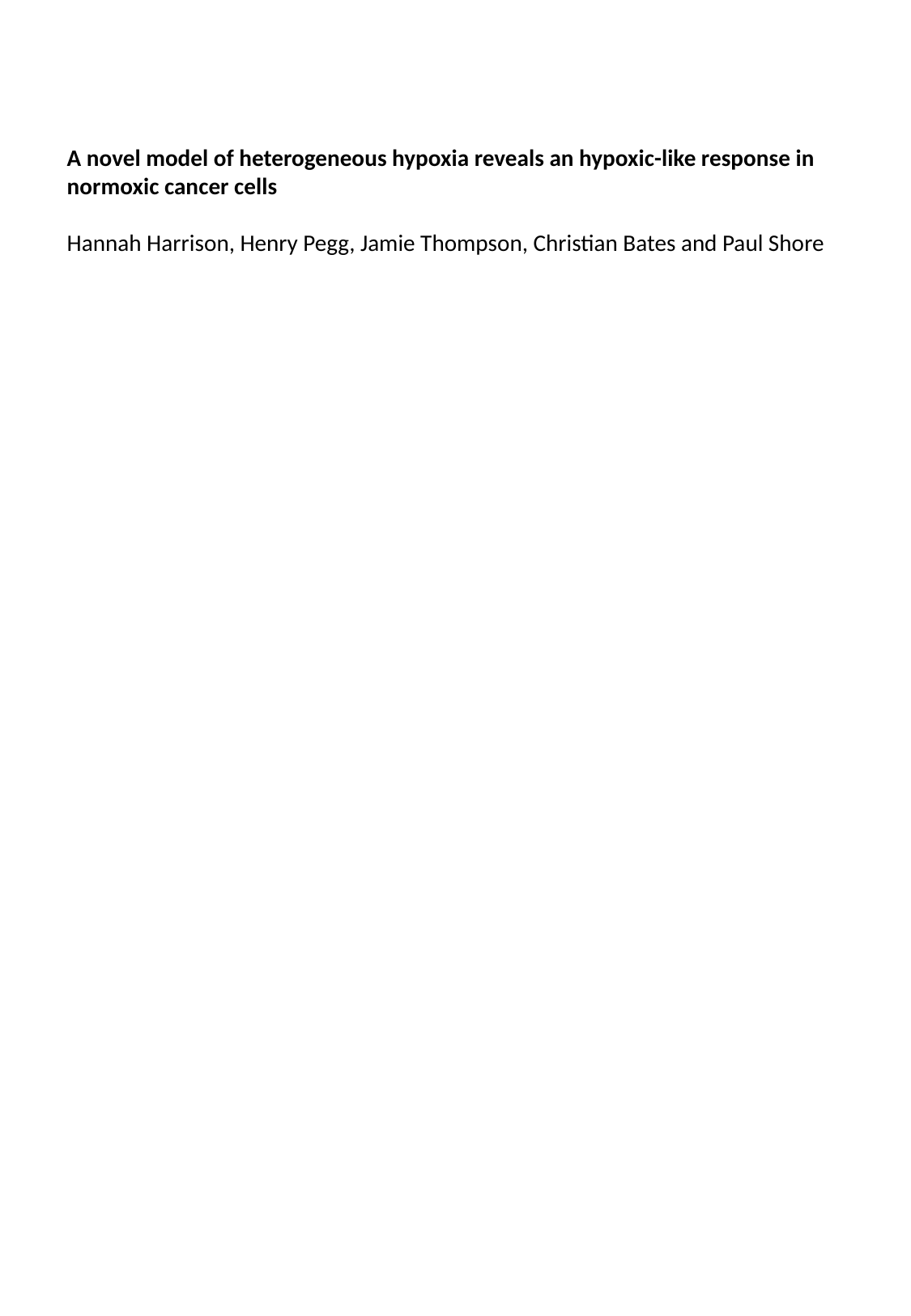

A novel model of heterogeneous hypoxia reveals an hypoxic-like response in normoxic cancer cells
Hannah Harrison, Henry Pegg, Jamie Thompson, Christian Bates and Paul Shore

## Slide 2
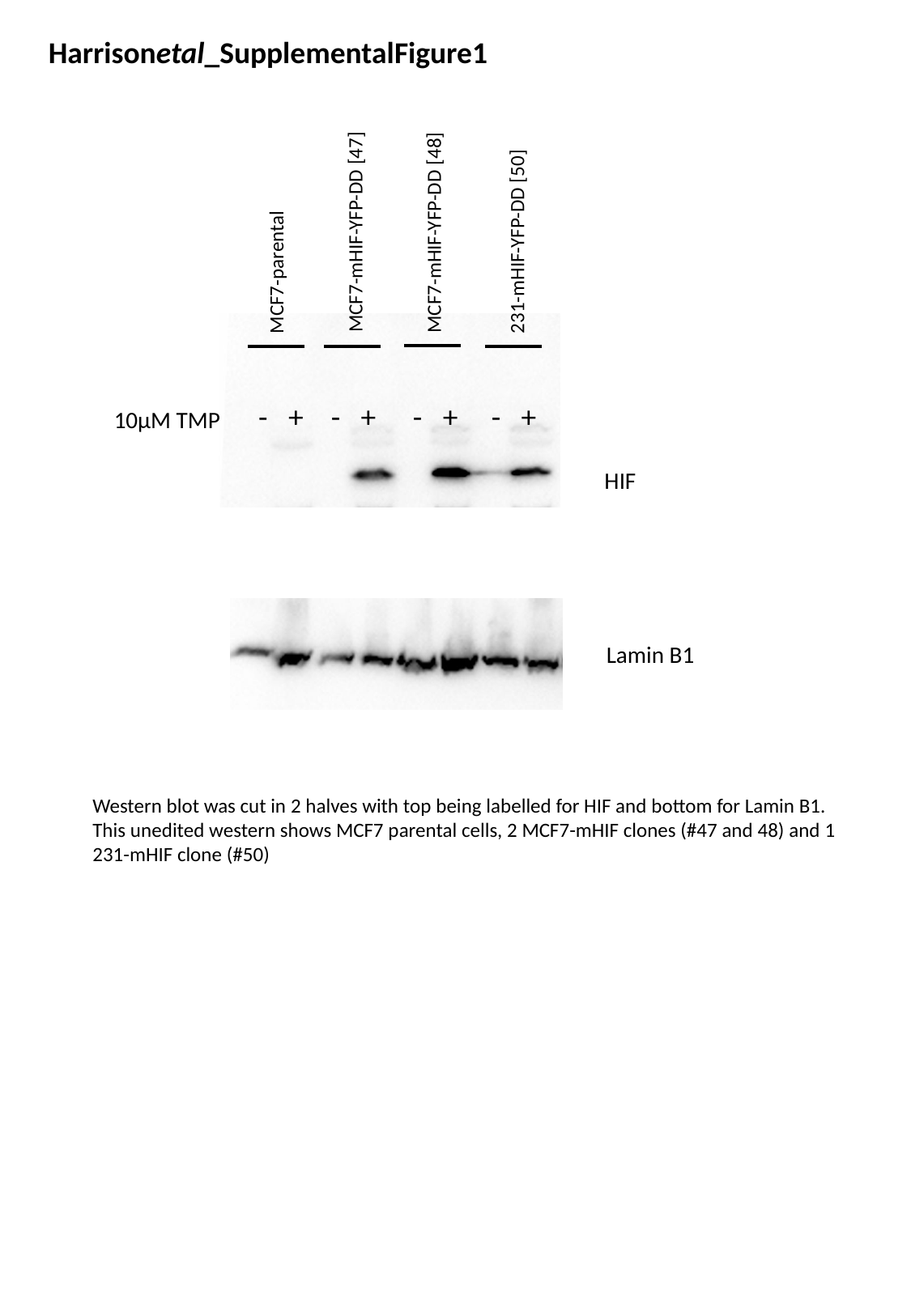

Harrisonetal_SupplementalFigure1
MCF7-mHIF-YFP-DD [47]
MCF7-mHIF-YFP-DD [48]
231-mHIF-YFP-DD [50]
MCF7-parental
- +
- +
- +
- +
10µM TMP
HIF
Lamin B1
Western blot was cut in 2 halves with top being labelled for HIF and bottom for Lamin B1.
This unedited western shows MCF7 parental cells, 2 MCF7-mHIF clones (#47 and 48) and 1 231-mHIF clone (#50)

## Slide 3
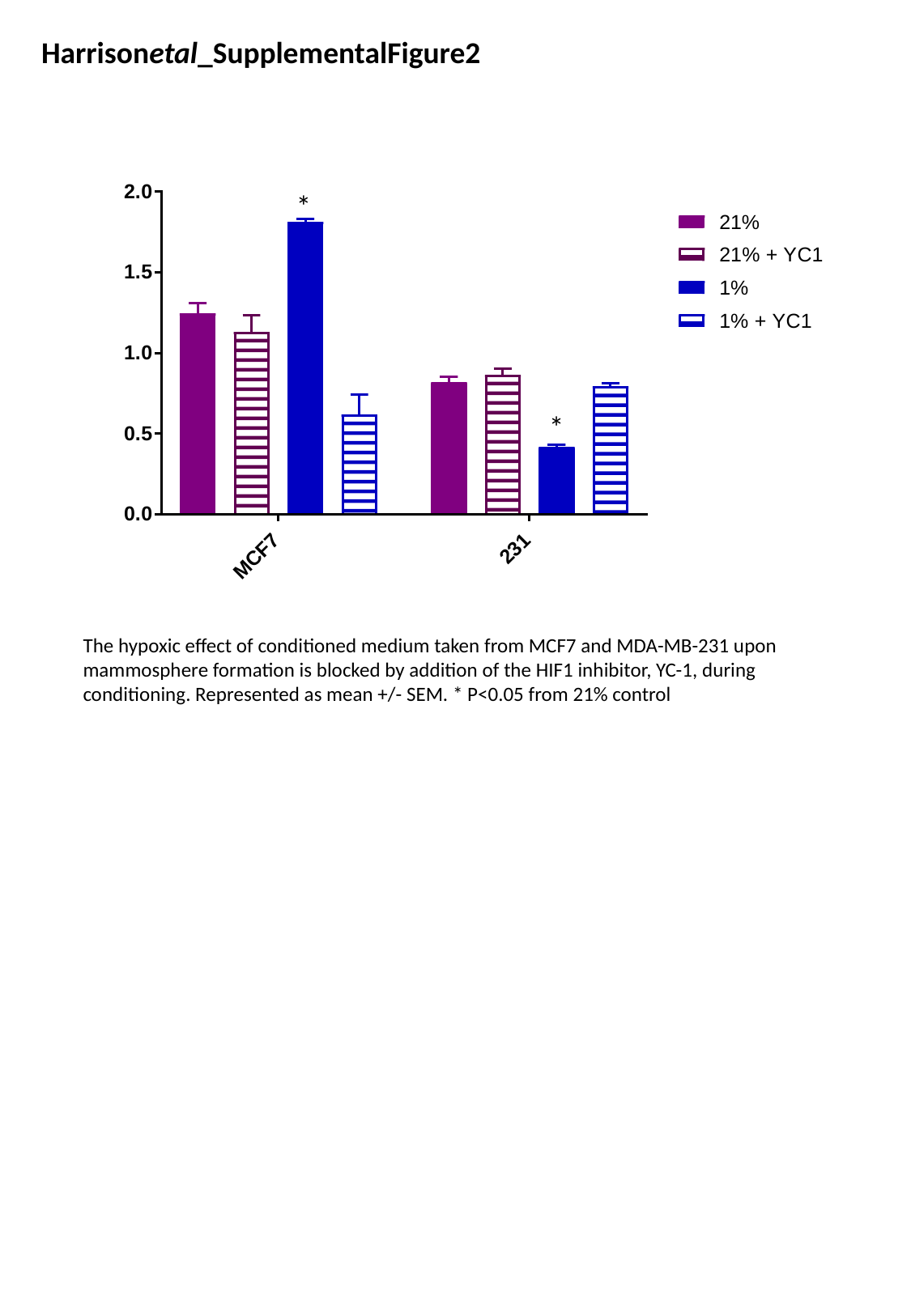

Harrisonetal_SupplementalFigure2
*
*
The hypoxic effect of conditioned medium taken from MCF7 and MDA-MB-231 upon mammosphere formation is blocked by addition of the HIF1 inhibitor, YC-1, during conditioning. Represented as mean +/- SEM. * P<0.05 from 21% control

## Slide 4
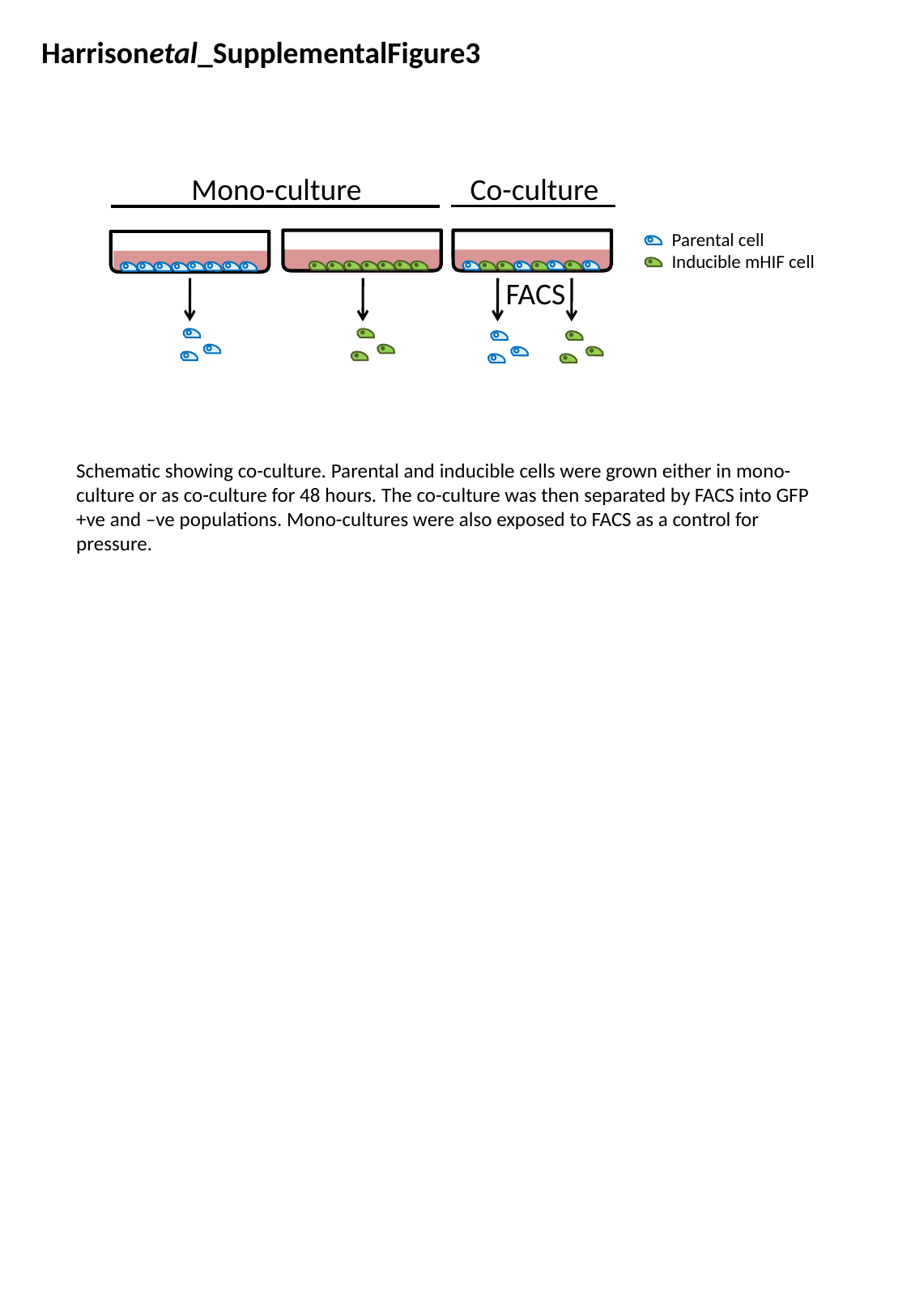

Harrisonetal_SupplementalFigure3
Co-culture
Mono-culture
Parental cell
Inducible mHIF cell
FACS
Schematic showing co-culture. Parental and inducible cells were grown either in mono-culture or as co-culture for 48 hours. The co-culture was then separated by FACS into GFP +ve and –ve populations. Mono-cultures were also exposed to FACS as a control for pressure.

## Slide 5
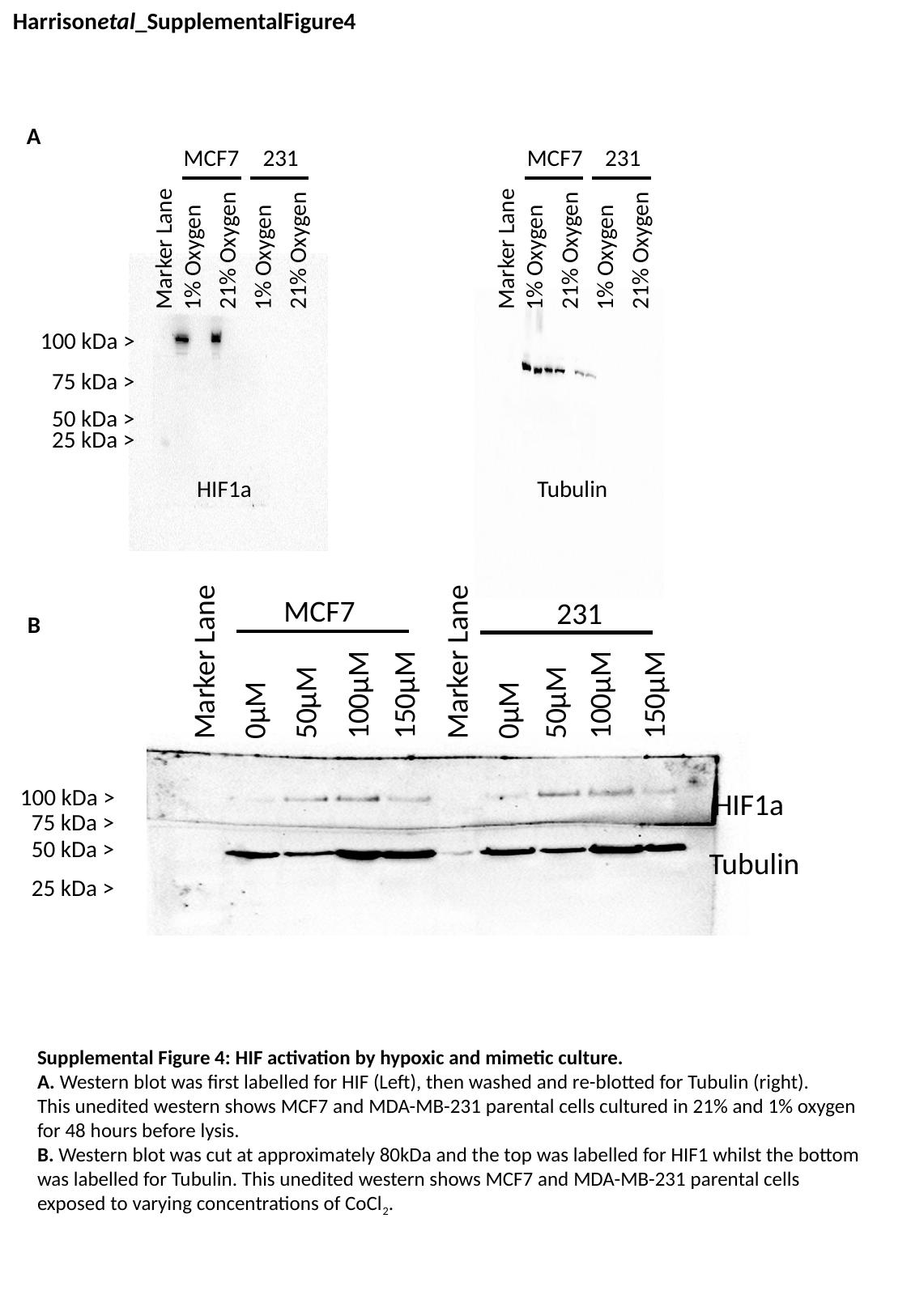

Harrisonetal_SupplementalFigure4
A
MCF7
231
MCF7
231
Marker Lane
Marker Lane
21% Oxygen
21% Oxygen
21% Oxygen
21% Oxygen
1% Oxygen
1% Oxygen
1% Oxygen
1% Oxygen
100 kDa >
75 kDa >
50 kDa >
25 kDa >
HIF1a
Tubulin
MCF7
231
B
Marker Lane
Marker Lane
100µM
150µM
100µM
150µM
50µM
50µM
0µM
0µM
100 kDa >
HIF1a
75 kDa >
50 kDa >
Tubulin
25 kDa >
Supplemental Figure 4: HIF activation by hypoxic and mimetic culture.
A. Western blot was first labelled for HIF (Left), then washed and re-blotted for Tubulin (right).
This unedited western shows MCF7 and MDA-MB-231 parental cells cultured in 21% and 1% oxygen for 48 hours before lysis.
B. Western blot was cut at approximately 80kDa and the top was labelled for HIF1 whilst the bottom was labelled for Tubulin. This unedited western shows MCF7 and MDA-MB-231 parental cells exposed to varying concentrations of CoCl2.
